# Diet and lifestyle factors associated with fish consumption in men and women: a study of whether gender differences can result in gender-specific confounding

**DOI:** 10.1186/1475-2891-11-101

**Published:** 2012-12-04

**Authors:** Maria Wennberg, Andreas Tornevi, Ingegerd Johansson, Agneta Hörnell, Margareta Norberg, Ingvar A Bergdahl

**Affiliations:** 1 Department of Public Health and Clinical Medicine, Occupational Medicine, Umeå University, Umeå 901 87, Sweden; 2 Department of Nutritional Research, Umeå University, Umeå, Sweden; 3 Department of Odontology, Umeå University, Umeå, Sweden; 4 Department of Food and Nutrition, Umeå University, Umeå, Sweden; 5 Department of Public Health and Clinical Medicine, Epidemiology and Global Health, Umeå University, Umeå, Sweden

**Keywords:** Fish consumption, Lifestyle, Gender, Confounding factors

## Abstract

**Background:**

Fish consumption and intake of omega-3 fatty acids from fish are associated with a lower risk of cardiovascular disease. However, a prospective study from northern Sweden showed that high consumption of fish is associated with an increased risk of stroke in men, but not in women. The current study aimed to determine if fish consumption is differently related to lifestyle in men compared with women in northern Sweden.

**Methods:**

Lifestyle information on 32,782 men and 34,866 women (aged 30–60 years) was collected between 1992 and 2006 within the Västerbotten Intervention Programme (a health intervention in northern Sweden). Spearman correlations coefficients (R_s_) were calculated between self-reported consumption of fish and other food items. Lifestyle variables were compared between fish consumption categories.

**Results:**

Fish consumption was positively associated with other foods considered healthy (e.g., root vegetables, lettuce/cabbage/spinach/broccoli, chicken, and berries; R_s_ = 0.21-0.30), as well as with other healthy lifestyle factors (e.g., exercise and not smoking) and a higher educational level, in both men and women. The only gender difference found, concerned the association between fish consumption and alcohol consumption. Men who were high consumers of fish had a higher intake of all types of alcohol compared with low to moderate fish consumers. For women, this was true only for wine.

**Conclusions:**

Except for alcohol, the association between fish consumption and healthy lifestyle did not differ between men and women in northern Sweden. It is important to adjust for other lifestyle variables and socioeconomic variables in studies concerning the effect of fish consumption on disease outcome.

## Background

The beneficial role of fish consumption or omega-3 fatty acids from fish on the risk of cardiovascular disease has been extensively studied. Most studies have concluded that fish consumption is associated with decreased risk of cardiovascular disease [1-3], although there is more evidence for myocardial infarction than for stroke [4]. However, fish consumption is associated with a healthy lifestyle in several populations and it can be argued that this protective association may be influenced by a healthy lifestyle. Most previous findings on fish consumption and lifestyle are from epidemiological studies that investigated the effect of fish consumption on disease outcome. The associations between fish consumption and other lifestyle variables have been described as baseline characteristics in these previous studies [5-8]. However, few studies have investigated the association between fish consumption and other lifestyle variables as the main aim of the study [9-11], and even fewer studies have reported if confounding by a healthy lifestyle differs between men and women [10]. We have previously observed results that raise concern for the possibility of gender differences in the northern Swedish population. In our prospective case–control study on the risk of stroke and fish consumption in northern Sweden, an increased risk of stroke was found in men reporting that they consumed fish more than three times a week [12]. In women, the tendency was the opposite, although this was not statistically significant. Therefore we hypothesize that male fish consumers in northern Sweden differ from female fish consumers with regard to confounding between dietary and other lifestyle factors, and that high fish consumption is a marker of otherwise unhealthy lifestyle in men in this population. We also speculate that lifestyle factors were not properly controlled for in our previous stroke study [12]. The relationship between fish consumption and lifestyle has not been thoroughly investigated in the population from northern Sweden.

The current study aimed to determine if there are gender differences in the association between fish consumption and other lifestyle variables in northern Sweden. Associations between fish consumption and self-reported intake of other foods and lifestyle variables were studied, in men and women separately.

## Methods

### The Västerbotten Intervention Program (VIP)

Health examinations of the population in the county of Västerbotten in northern Sweden have been continuously conducted since 1985 within the VIP [13], which is an intervention concerning risk factors for cardiovascular disease and diabetes. All citizens in Västerbotten are invited to participate in a health examination at their healthcare center when they turn 40, 50 and 60 years (also at 30 years until 1995). The participants fill out an extensive questionnaire on lifestyle, including a food frequency questionnaire (FFQ), a medical examination is carried out, and participants are asked to give blood samples for future research. The participation rates have increased from approximately 55% in the early 1990s to 65% from 2004–2006. Dropout analyses, on data from 1992–1993, indicated only small differences between participants and non-participants regarding social and health factors, indicating that the selection bias was small [14]. In total, 32,782 unique men and 34,866 unique women participating in the VIP between 1992 and 2006 were enrolled in this study, after exclusion of individuals with a food intake level (FIL = reported caloric intake/basal metabolic rate) below the 5^th^ or above the 97.5^th^ percentile [15].

### The FFQ

Changes have been made to the VIP FFQ over time, and to simplify data handling, only individuals filling out the optically readable 64-66-item FFQ used since 1992 were included in this study. An 84 item version of the FFQ has been described by Johansson et al. [16]. The FFQ has been validated against 10 24-hour dietary recalls [16] and the questions on fish consumption were validated against erythrocyte levels of eicosapentaenoic acid (EPA) and docosahexaenoic acid (DHA) [17]. Fair correlations (R_s_ = 0.42-0.51, p ≤ 0.005) were found between the intake of EPA and DHA according to the FFQ and levels of erythrocytes. In the 64–66 item FFQ, some foods in the 84 item version were combined into groups to obtain a shorter FFQ. The FFQ has 9 answer alternatives ranging from “never” to “4 times a day or more often” [16]. The answers were transformed to the number of intake/day or intake/week.

The questions asked regarding fish consumption in the present study were as follows; “How often do you eat lean fish (e.g., perch and cod)?” and “How often do you eat fatty fish (e.g., herring, lavaret, and salmon)?” The intake frequencies of lean and fatty fish were calculated as times/week of total fish. These questions on fish consumption were consistent in the different versions of the FFQ.

The questions asked on fruit and berries (apple/pear/peach/citrus fruit, bananas, and berries) were combined into total intake/week. Likewise, a vegetable variable was created by combining root vegetables, lettuce/cabbage/spinach/broccoli, and frozen vegetables. Because of differences over time in the FFQ concerning questions on fruit and vegetables, there were fewer observations for these variables. An alcohol variable was created by combining the intake frequencies of strong beer (beer with more than 3.5% of alcohol), wine and spirits to intake/week, but these items were also considered separately.

### Lifestyle and socioeconomic variables

Smoking habits, educational level and physical activity were included in the questionnaire. Smoking was categorized as non-smokers (including ex-smokers and occasional smokers) or current smokers. Educational level was categorized as academic education (e.g., completed a university degree) or no academic education. Those subjects who reported that they never exercised or took a walk less than twice a week were categorized as physically inactive.

### Statistical analysis

Mean levels of dietary and other lifestyle variables of interest were compared between those subjects who reported total fish consumption > 3 times/week (high consumers) and those reporting ≤ 3 times/week (low to moderate consumers) within each gender. This categorization was used to enable investigations on characteristics for those with an unusually high consumption of fish. To address differences between men and women, the percentage differences across these groups were compared and tested for significance using t-tests. In these calculations, mean levels in the low to moderate consumers were considered as the reference group in each gender. Comparisons of mean levels of dietary and lifestyle variables were also performed with fish consumption in the following categories: < once a week, 1–3 times/week, and > 3 times/week. Spearman correlation coefficients were calculated between the frequency of fish consumption and other food items, in men and women separately. Spearman correlation coefficients were also calculated for age groups (<40, 40–49, 50–59 and ≥ 60 years) and time periods (1992–1996, 1997–2002 and 2003–2006) to determine if the results were consistent.

### Ethics

All participants gave informed consent and the study was approved by the Regional Ethical Review Board in Umeå.

## Results

For both men and women, high consumers of fish were older, had a higher reported consumption of vegetables, fruit and berries, a higher level of physical activity, and a higher educational level than low to moderate consumers (Table 1). Men who consumed a lot of fish smoked less than low to moderate consumers. Male and female high consumers of fish had a higher consumption of wine. There were gender differences for consumption of strong beer and spirits, with a higher consumption in male high consumers of fish than in low to moderate consumers, while women who consumed a lot of fish reported a lower consumption of strong beer than female low to moderate consumers. Differences between high consumers of fish and low to moderate consumers were larger for men compared with women concerning the reported consumption of fish and alcohol (all varieties) and the proportion of physically active subjects, smokers, and the proportion of subjects with an academic education.

**Table 1:** Lifestyle characteristics for the population in the Västerbotten Intervention Program, by fish consumption level

		**Fish ≤ 3 times/week**		**Fish > 3 times/week**				
		**N**	**Mean (95% CI)**	**N**	**Mean (95% CI)**	P-value*	**Percentage difference**	**P for non-difference between men and women†**
Age (y)	Men	31662	47.4 (47.3, 47.5)	1120	51.2 (50.8, 51.7)	< 0.001	8.17	0.396
	Women	33172	47.3 (47.2, 47.4)	1694	51.4 (51.0, 51.8)	< 0.001	8.79	
Lean fish	Men	31662	0.57 (0.56, 0.57)	1120	2.12 (2.04, 2.21)		275	0.020
(times/week)	Women	33172	0.65 (0.65, 0.66)	1694	2.34 (2.28, 2.41)		258	
Fatty fish	Men	31662	0.48 (0.48, 0.48)	1120	1.92 (1.81, 2.02		298	<0.001
(times/week)	Women	33172	0.50 (0.49, 0.50)	1694	1.58 (1.52, 1.65)		220	
Vegetables	Men	19778	5.05 (4.99, 5.12)	746	8.80 (8.29, 9.31)	< 0.001	74.0	0.265
(times/week)	Women	19874	8.38 (8.29, 8.47)	1138	13.4 (12.9, 14.0)	< 0.001	60.4	
Fruit & berries	Men	19778	8.11 (8.01, 8.20)	746	11.9 (11.3, 12.5)	< 0.001	46.8	0.197
(times/week)	Women	19874	12.7 (12.6, 12.9)	1138	16.7 (16.1, 17.2)	< 0.001	30.7	
Alcohol	Men	31662	1.35 (1.33, 1.36)	1120	1.62 (1.50, 1.74)	< 0.001	20.1	0.014
(times/week)	Women	33172	0.94 (0.93, 0.95)	1694	0.98 (0.92, 1.04)	0.190	3.95	
Strong beer	Men	31662	0.47 (0.46, 0.47)	1120	0.55 (0.47, 0.62)	0.043	17.0	<0.001
(times/week)	Women	33172	0.23 (0.23, 0.23)	1694	0.20 (0.19, 0.21)	< 0.001	−13.1	
Wine	Men	31662	0.48 (0.47, 0.48)	1120	0.63 (0.57, 0.68)	< 0.001	31.3	<0.001
(times/week)	Women	33172	0.49 (0.49, 0.50)	1694	0.56 (0.52, 0.60)	0.002	13.2	
Spirits	Men	31662	0.40 (0.40, 0.41)	1120	0.44 (0.41, 0.48)	0.022	10.4	0.016
(times/week)	Women	33172	0.22 (0.21, 0.22)	1694	0.22 (0.20, 0.24)	0.393	0.92	
Physically active	Men	30912	76 (75, 76)	1097	85 (83, 87)	< 0.001	12.0	0.0019
(%)	Women	32638	84 (84, 84)	1661	90 (88, 91)	< 0.001	7.13	
Smokers	Men	31214	25 (25, 26)	1105	21 (19, 24)	0.003	−15.3	<0.001
(%)	Women	32931	26 (25, 26)	1684	25 (23, 27)	0.263	−3.68	
Academic education	Men	31526	22 (21, 22)	1115	27 (25, 30)	< 0.001	24.3	0.0018
(%)	Women	33006	30 (29, 30)	1670	34 (31, 36)	< 0.001	12.7	

*P-value for non-difference between low to moderate and high fish consumers within gender.

†P-value for equal percentage difference between men and women between low to moderate and high fish consumers (2-sample t-test).

**Figure 1:**
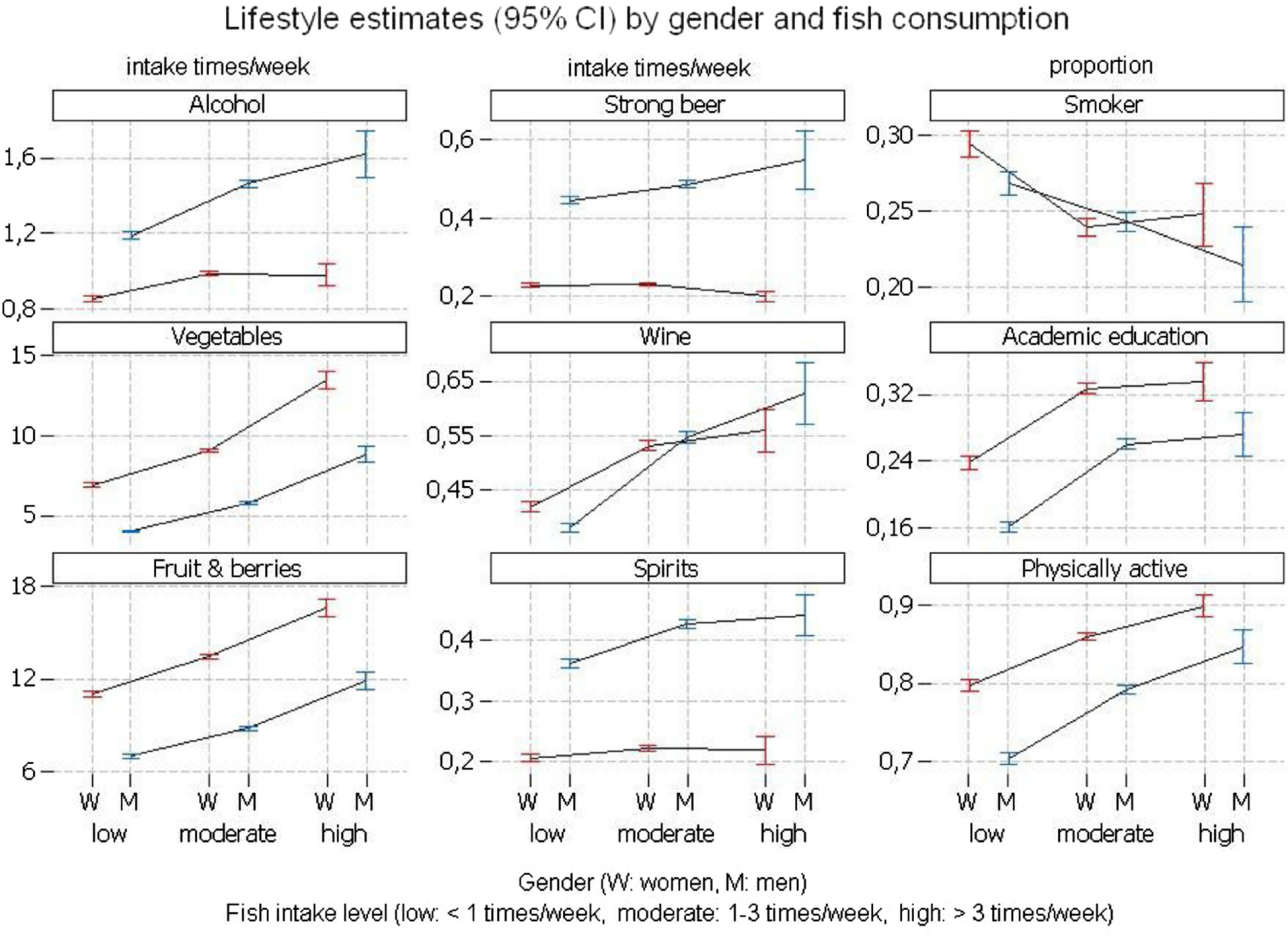
Estimates of lifestyle characteristics by gender and fish consumption (in 3 levels). Fish intake refers to total intake of fatty and lean fish.

When fish consumption was classified into 3 categories (< once a week, 1–3 times/week, and > 3 times/week), fish consumption was generally associated with a healthier behavior. Also when comparing the medium consumption category (1–3 times/week) with the low consumption category (< once a week), higher fish consumption was associated with healthier behavior for both men and women. Low fish consumption was associated with a higher proportion of smokers also in women (Figure 1).

**Figure 2:**
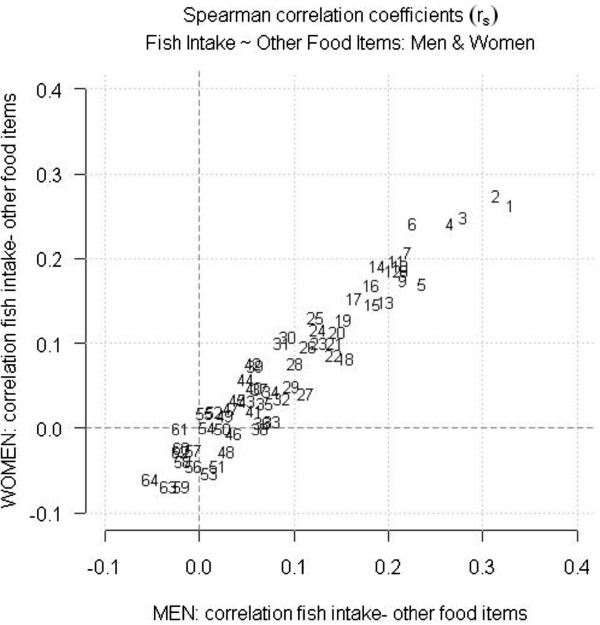
**Gender-specific correlations between consumption of fish and other foods according to the northern Sweden food frequency questionnaire. **The horizontal axis shows correlations between fish consumption and other foods in men. The vertical axis shows the corresponding correlations for women. High positive values correspond to strong correlations with fish consumption. 1.salty fish 2.rootvegetables 3.lettuce/cabbage/spinach/broccoli 4.chicken 5.tomato/cucumber 6.berries 7.boiled potato 8.beef stew 9.smoked fish 10.apple/pear/peach/citrus fruit 11.brown beans 12.fibre bread 13.steak 14.cooking oil 15.fibre cereal 16.porridge 17.oil- and vinegar dressing 18.wine 19.rice 20.low alcohol beer 21.bananas 22.cream 23.minced meat 24.egg dishes 25.soured milk (0.5% fat) 26.meat on bread 27.bacon 28.tea 29.ice cream 30.fibre crisp bread 31.milk (0.5% fat) 32.soured milk 33.spirits 34.fruit soup 35.medium alcohol beer 36.potato dumpling/pancake 37.liver pâté/sausage on bread 38.hard cheese (28% fat) 39.water 40.spagetti 41.cookies 42.hard cheese (10-17% fat) 43.sweet buns 44.butter in food 45.filter coffee 46.sausage 47.low fat margarine on bread 48.beer 49.corn flakes 50.margarine on bread 51.candy 52.butter on bread 53.margarine in food 54.milk (3% fat) 55.sugar/jam 56.fried potato 57.pizza 58.hamburger 59.crisps/snacks 60.soda 61.milk/soured milk (1.5% fat) 62.butter based margarine 63.white soft and crisp bread 64.boiled coffee The numbering is ordered by the magnitude of correlation on the horizontal axis. Fish intake refers to total intake of fatty and lean fish.

When Spearman correlation coefficients were calculated for total intake of fish and other dietary variables, we found that the associations between fish consumption and the intake of other foods were similar in men and women (Figure 2). Reported fish consumption was positively correlated with other foods considered healthy (e.g., root vegetables, chicken, berries, and lettuce/cabbage/spinach/broccoli) and was poorly correlated with many foods considered unhealthy (e.g., crisps/snacks, white bread, fried potatoes, pizza/hamburger, and soda). The same plots restricted by age group or time period showed similar correlations (data not shown).

Because alcohol consumption was the only factor that was differently associated with fish consumption in men and women but was not accounted for in the statistical analysis in our previous study of stroke and fish consumption [12], we made a new analysis of those data, now including alcohol in the multivariate model. This did not change the point estimate for the stroke risk in relation to fish consumption (without alcohol: OR: 1.24 [95% CI: 1.01, 1.51]; with alcohol: OR: 1.23 [95% CI: 1.00, 1.52] for intake of fish, meals/week, in men).

## Discussion

The current study found that fish consumption was positively associated with a healthy lifestyle in both men and women in a large study of the adult population in the county of Västerbotten in northern Sweden. This is in agreement with previous studies [8,10]. We did not find any indication of gender differences, except for alcohol.

There are limitations concerning the dietary measurement method used in this study. FFQs are designed to rank participants according to their dietary intake, not to measure the whole diet. Therefore, we only reported correlations between intake frequencies in this study, not absolute food consumption. The fish consumption questions used in the FFQ have been validated using erythrocyte levels of EPA and DHA, which is a strength of the current study. Spearman correlation coefficients between estimated intake of these fatty acids according to the FFQ and erythrocyte levels were satisfactory (R_s_ = 0.42-0.51) [17].

A problem in dietary surveys is that people often report a more healthy behavior than what is actually true [18]. It is well known in the population of northern Sweden that fish is a healthy food. This might increase the positive association between fish consumption and other foods considered healthy and other healthy behaviors. In addition, underreporting is a problem in dietary surveys. In a previous study, no gender difference in the prevalence of underreporting in the northern Swedish population was found [19], but it is unknown if there is a difference in what men and women underreport, which is a limitation. In the current study, people below the 5^th^ and above the 97.5^th^ percentile of food intake level were excluded, to avoid an effect from the most evident under- and overreporters.

Our finding of higher consumption of alcohol in male high consumers of fish is interesting when considering our previous finding of a higher risk of stroke in male high consumers of fish in northern Sweden [12]. High blood pressure is the dominant risk factor for stroke and high consumption of alcohol is positively associated with hypertension. However, light to moderate alcohol consumption may be protective against ischemic stroke [20]. Hypertension, but not alcohol consumption, was adjusted for in our previous stroke study [12].

The finding of a positive association between alcohol and fish consumption in men, in this larger study performed in the same population as our previous stroke study [12], called upon additional analysis in our previous stroke study. Therefore, we requested complementary information on alcohol consumption (frequency of strong beer, wine and spirits consumed) in our previous stroke study. However, addition of alcohol in the statistical model did not change the point estimate for the stroke risk in relation to fish consumption. Therefore, the finding of a higher risk of stroke in men reporting high fish consumption could not be explained by a higher intake of alcohol. The hypothesis that high fish consumption is a marker of unhealthy lifestyle in men in this population must be rejected. As regards the factors measured in this study, there are no gender-specific confounding factors in the association between fish consumption and the risk of stroke in northern Sweden. Additional studies are warranted to clarify the finding of an increased risk of stroke in men reporting high consumption of fish in northern Sweden.

## Conclusions

Fish consumption is positively associated with other healthy lifestyle factors (consumption of vegetables and fruit, exercise, and not smoking) and a higher educational level in both men and women in northern Sweden. It is important to adjust for other lifestyle variables and socioeconomic variables in studies concerning health effects of fish consumption.

## Competing interests

The authors declare that they have no competing interests.

## Authors' contributions

MW participated in the design of the study, the acquisition of data, interpretation of data, and drafted the manuscript. AT participated in the design of the study, performed the statistical analysis, and participated in interpretation of data. IJ participated in the acquisition of data and interpretation of data. AH participated in the design of the study and interpretation of data. MN participated in the acquisition of data and interpretation of data. IAB participated in the design of the study and interpretation of data. All authors read and approved the final manuscript.

## Disclaimer

The paper reflects only the authors′ views: the European Union is not liable for any use that may be made of the information.
